# The Relations of the Nursing Profession to That of Medicine and to Society

**Published:** 1904-09

**Authors:** 


### THE RELATIONS OF THE NURSING PROFESSION TO THAT OF MEDICINE AND TO SOCIETY.*

Plato, so the fables have it, when an infant in the cradle, was visited by some bees that tarried a moment, leaving not a sting,

*An address by Charles A. L. Reed, A.M., M.D., Cincinnati, delivered at the Annual Commencement of the Training School of the Miami Hospital, Dayton, O., for 1904.

but some honey upon his lips. It was this circumstance that accounted for the facility of phrase which characterized his eloquence in subsequent life. My own personal recollection of contact with bees is so distinctly painful and so far removed from any suggestion of sweetness that I find it impossible to express in honeyed phrase my appreciation of this opportunity to address so large, interested and benevolent an audience. This defect on my part is all the more embarrassing when I realize that the object of this occasion is to confer a distinction upon the young ladies who comprise the graduating class. As I glance at them for a moment I am reminded of an intimation that I received on the occasion of a recent visit to your city, to the effect that a new rule had been established for the admission of pupils to this institution—a rule which consisted in nothing more or less than that all applications should be made by photograph. It was, furthermore, intimated that the final selection was made one of the prerogatives of the staff, a circumstance which, doubtless, accounts, in large part, for their zealous devotion to duty. If this fact, however, is brought to my mind by the presence in which I find myself, there are still other reflections that are prompted by the circumstances of the occasion. Thus, for instance, while we recognize this graduating class as the essential feature of the evening, we must recognize, also, that this audience, assembled under these circumstances, and with this object in view, has a special significance. I think we may best arrive at an understanding of the meaning underlying both of these factors if we pause for a moment to reflect that the American life of to-day presents two features—very distinct features—of development. The one is a rapid evolution of groups of people who are devoted to a common but special calling, each group, according to its educational basis and according to the completeness with which it absorbs the energies and activities of its members, being designated as a profession. This tendency to specialization is spoken of by writers as the chief social characteristic of the age. These groups, as rapidly as they are formed, become really distinct bodies. As a rule, the individual members assemble and effect some kind of organization for the selfish purpose, if you please, of protecting their personal interests and for the altruistic purpose, let us not doubt, of developing their educational basis and their social and legal status. I allude to these circumstances now because we, to-night, stand formally in the presence of one of these newly evolved groups, one of these newly developed professions, and the circumstance is one which suggests certain rather important reflections. What is this new profession? Whence came it? What place is it to occupy in society and in the State?

Like most new developments, the nursing profession has had its origin in the actual necessities of society. We look back through the ages and are thrilled at the kindly offices of the Good Samaritan, and we bow with reverence at the memory of the sacred function of Mary. These examples, however, of personal benefactions, if carefully analyzed, will be found to have secured their great applause only because they contrasted so strikingly and so mercifully with the ordinary conduct of mankind at that period. The act of the Good Samaritan, considered without reference to its motives, would to-day prove to be but crude succor, while the tender mercies of Mary would be accepted but as ordinary manifestations of maternal duty. The gentleness no less than the rudeness of knight-errantry, the chivalric regard for each other's welfare, were the redeeming features of an age that must, otherwise, stand on the records as barbaric alike in its motive and in its method. Attendance upon the sick in the hospitals of the Middle Ages was invariably in the hands of either the monks or the hirelings of the State. One pauses with a shudder to think of the sufferings of the individual in the horde-like armies of Cyrus, of Alexander, and of Hannibal. Thus it went through the centuries from bad to worse, until the awful days of the Crimea. It was then that a woman, English by parentage, but born under the genial suns of Tuscany, a woman who had already entered an institution of the Sisters of Mercy, feeling the grand impulse to succor the wounded and heal the sick and to minister with gentle mercy to the suffering soldiers, wended her way to the Orient. It was a happy day when the smiling face of Florence Nightingale, with her ninety-two loyal assistants, appeared in the dismal wards of the military hospital at Scutari. It was this influence, inaugurated thus with dramatic effect, in 1854, that sent through all modern society the impulse to improve, as Florence Nightingale had improved in the Crimea, the administration of the hospitals. But things move slowly. It would seem, indeed, as if anything so demonstrably beneficent as this movement would have been taken up by society at once and adopted whenever the opportunity presented itself. It was just fifty years ago, however, this very spring that the initial step was taken. The army organized that year at Scutari has grown slowly but steadily, has embraced country after country, until to-day—to-night—we are extending our greetings to its recruits here in Dayton.

It is not my purpose to discuss the history of the evolution of this good profession. We may, however, mention the significant fact that in England, where the movement received its first, although tardy, recognition, it has passed through various vicissitudes. In the first place, it was relegated to the purely charitable orders, the members of which, without particular training, simply devoted themselves, in the fullness of their hearts and in the charity of their souls, to the welfare of mankind. Male nurses were still features of hospital administration in both the metropolitan and provincial institutions of that country, as they were, indeed, all over the world, while the few women employed as nurses were too generally of the type of Sairy Gamp. There came, however, a new revelation to the medical profession. It was the dawn of a new era. This revelation came in the form of the discovery that invisible organisms, to be detected only by microscopes of the highest power, were responsible for many so-called medical diseases, and for practically all of the serious and previously fatal complications attending surgical conditions. That there was an invisible foe to be found and that there were insidious enemies lurking everywhere—on bed-linen, on dresses, on instruments, on door-knobs, and particularly upon the hands of doctors and attendants—became a demonstrated fact. To find this foe required a contending army, and it has gradually been brought into the field of action. It is recruited, drilled, and, as we are doing here to-night, commissioned.

What then are we to recognize as the proper relation of this army, of this new profession, to society in general? In the first place, the trained nurse as we find her to-day is an educated woman, who, as a rule, comes from refined families. She enters upon her new calling most generally for altruistic reasons, for motives which prompt tender womanhood to minister to want and suffering. She enters the training-school where she receives instruction which embraces the careful perusal of text-books of high scientific grade. She listens to lectures by able physicians who explain to her the philosophy of disease and make her familiar with the enemies against which she has to contend during the rest of her life, and then, finally, this is all exemplified in the ward, where the teaching is carried into practical effect under the direct supervision of those already drilled in the calling. These facts being true, it follows that we must recognize this profession as an educated profession, and accord to it the position and prerogatives that go with culture and refinement, that go with benevolence and with a high grade of usefulness.

PRESENT RECOGNITION AND ACTIVITY OF THE PROFESSION.

There are, however, some additional thoughts that press themselves to the front. If it is true that the profession of nursing stands really upon such an educational basis, if it is true that it is thus distinctly evolved as a factor in society, how is society to avail itself of the services of this profession, how protect itself against the unfortunate ministrations of those who are unqualified to fulfill its requirements? It is true that inquiry may be made of the individual applicant, but it is equally true that the individual applicant may give some very misleading replies. It follows, therefore, that this profession, in the interests of the public, should be given certain legal protection, a certain definite legal status. The medical profession has moved in its own behalf in this direction, the dentists have done the same, so have the veterinarians and the undertakers. In several of the States there are now pending bills which, if passed, will prescribe certain specific requirements for admission to this new profession, a definite curriculum will be prescribed as essential to graduation, certain examinations after graduation will be prescribed and enforced as conditions to secure a license. If this principle can be established with regard to nursing, this principle of licensure by the States, society will be in a position to avail itself intelligently of the services of those, who, presumptively, are qualified to discharge the duties. It seems, however, that in Ohio there is a constitutional provision by which women can not be appointed to any executive office, and that, therefore, if an attempt were to be made to establish a State board for the purpose of examination and licensure of trained nurses the board would have to consist of men. Of course, this proscriptive policy is but another reminder of the dark ages, and must sooner or later yield to the diffusion of intelligence and to the general broadening of our humane impulses. In the meantime, however, it exists as an actual condition, and interferes with the theory that I have been advocating, and that must sooner or later command recognition in every State in our country. What, then, ought the nursing profession to do for itself, and what ought society to do in its own behalf concerning this profession until such time as public opinion in Ohio shall have advanced to that point that women, in whatever calling, may be given the rights to manage their own affairs ? You will observe that I am proceeding upon the principle that the nursing profession is essentially a woman's profession, and that as a profession it ought to have the right to manage its own affairs, prescribe its own membership and control its own licensure. This it can do now only through the means of voluntary organization. Associations, chartered, if you please, by the State, should be established, with branch or constituent societies in each of the important centres of population, in which the conditions of membership should be carefully specified and rigorously enforced. Society would do well to take the firm ground that none but members of such association should secure recognition and employment.

This brings me to a final, although, possibly, somewhat indelicate phase of this question, namely, that of compensation. Professor Hicks, in his recent masterly work on the "Theory of Economics," has emphasized the fact that professional skill rendered to a community is distinctly a commodity, having as intrinsic a money value as the merchandise that the dealer takes from his shelves. This being true, and I believe that nobody in this day will question the accuracy of the observation, it follows that the question of compensation, determined according to the general law of values, is one that requires careful consideration. It is interesting sometimes to consider just what in the matter of value is involved in the question of professional services. What are the elements that give to it a certain money value? In the first place, we must recognize the time and money spent in the acquisition of knowledge as so much cash capital. In the next place, we must recognize the time and the labor involved in rendering services to the recipient, and finally, we must take into account the elements of responsibility incurred by the one who renders such service. Each of these elements might with propriety become the theme of prolonged discussion, but time will not permit. I will call attention to two of them—the question of value to the recipient of the services and the question of responsibility on the part of the one who renders them. It is something of importance to the individual afflicted with either a disease or an injury to be protected against the influences that lurk as a constant menace to his life. Such guardianship may, and frequently does, possess distinct life-saving value. What, then is it worth in dollars and cents to the individual to have his life saved? Of course, to push this question to its final conclusion would be to extol professional services to such a point that, to receive them, would involve the payment of not a fee, but a ransom. This, of course, would be as great an evil as the other; for all charges for services ought to be made with reference to the patient's other existing obligations in life. This relationship is one that must enter largely into the determination of values, and yet, for practical purposes, it is important that there should be a certain recognized and conventional compensation. This, as a matter of fact, exists, and no complaint ought to be entered at this conventional compensation, justified as it is by the high character of the services rendered.

Now I want to turn to the other element that is represented here this evening, namely, the general public. What prompts the public, as represented in this distinguished and cultivated audience, to gather here to-night? I may be pardoned if I answer my own question by saying that this is but a manifestation of what, the world over, must be recognized as American altruism. I say American because I believe in this country this particular way of doing this particular thing is exemplified as it in no other country. It is interesting to note what has been done in the way of benefactions in this country. A recent writer on this subject inaugurated a systematic investigation for the purpose of determining the general aggregate of benefactions, excluding all gifts of less than $5,000. The result of his inquiry, extending over a period of ten years, showed that the aggregate of gifts varied from $27,000,000 to $107,000,000 annually, while the grand aggregate for the entire decade amounted to $610,410,000. If, now, we remember that charitable offerings that are given in small sums of less than $5,000 probably exceed the grand aggregate of those given in sums of that or even larger figures, we shall find that, covering the period of ten years from 1893 to 1903, the American public has given for religious, charitable and educational purposes considerably in excess of a billion dollars. The results are that our universities are possessed of the best buildings, our professors are given the best salaries, our hospitals are the best constructed and are the best equipped of any in the world.

I have mentioned this grand aggregate to call attention to the amount of charitable work that finds its expression in dollars and cents. I wish, however, on behalf of my own profession, to call your attention to another phase of this subject. If, now, you will remember that services rendered to hospitals the country over are entirely, or at least for the most part, gratuitous, and if you will compute the number of visits made by the members of hospital staffs, and compute them at the rate charged for the same services in private life, you will find that the amount given by the medical profession alone, for a single year, is in excess of that given by the opulence of the country for the entire period of ten years. In short, the medical profession, through the avenue of services, gives to public charity ten times as much as that given by the combined wealth of this the wealthiest country of the world. This is not mentioned in a boastful spirit, and I doubt if any member of this audience has previously heard the thought advanced by a single physician in the country. The services are rendered cheerfully, with no hope of compensation, other than that which comes from the happiness of the doing. But would a member of the legal profession try a case for the community without compensation? Would members of the legal profession accept a clientele without money and without price, simply because that clientele happened to be under a roof supported by charity? I indulge in no reflections upon the generosity of our brethren of the law; I simply draw a contrast to emphasize just what is being done in this community, for instance, in the way of bearing a burden that poverty and misfortune impose upon every country. This altruism on the part of the medical profession goes even farther; it finds expression in the very existence of this new profession, whose ranks are being recruited here to-night. But, happily for our blessed country, the motive of generosity, the motive of benevolence, is not restricted to any profession or to any calling. This fact finds ample illustration in the money given and in the labor bestowed in building up the splendid, the model institution that to-night stands sponsor for the professional existence of these young ladies. I am free to say that in observations that have embraced many cities and more than one country of the world, I have nowhere seen more adequate facilities for the care of the sick than those offered by the Miami Valley Hospital of Dayton. For these splendid results the community is indebted not only to the benevolence of its more opulent members, but particularly to the self-sacrificing devotion of the ladies who comprise the board of managers. They are entitled not only to the gratitude of rich and poor alike, but should receive, as I am assured they do receive, the cordial support of every agency that may broaden their influence and promote their charitable enterprise. Among these agencies are to be counted in particular the pulpit and the press. Religion, to-day, to be effective, must appeal to the practical helpfulness of the masses. This fact is recognized and acted upon by the clergy, who, with their medical confreres and with this newer profession, are to be recorded among the altruistic agencies of the community. The press, too, is ordinarily generous in the support of charitable movements. I say "generally" for the reason that within the last ten days I have encountered the first exception to the rule. It was at the beautiful little city of Austin, Texas, where, with a population not a tenth of that of Dayton to draw upon, the ladies have built two excellent hospitals, which, while much smaller than those of Dayton, still, in point of excellence, are entitled to rank in the same class. I had occasion to operate in both of them, and naturally expressed my surprise and delight at the facilities that were afforded me. One of the ladies thought that it would help them in their struggle if I would say to the community what I had said in private. She accordingly requested a representative of the local paper to call upon me. When he found what was wanted, he said: "They want a free write up, do they? Well, they won't get it. Nothing, nothing goes into this paper that ain't paid for!"

When I expressed my surprise that a newspaper should take such a position toward the charitable movements in the community upon which it depended for its existence, he simply replied: "Well, it don't go!"

I don't know whether it "went" or not, for I have never since seen an issue of the paper. But if I could control the ladies of Austin I would see to it that their husbands, fathers, brothers and others engaged in business stopped abruptly and finally every vestige of advertising patronage to that paper until its managers had the good heart to bear a proper share of the charitable burdens, or the good sense to leave the community.

I only mention this to emphasize the fact that here in Dayton, as in every other community, outside of Austin, of which I have any knowledge, the cordial support of the press in movements such as these is something to be appreciated.

